# Three-Dimensional Voxel-Wise Quantitative Assessment of Imaging Features in Hepatocellular Carcinoma

**DOI:** 10.3390/diagnostics13061170

**Published:** 2023-03-18

**Authors:** Chongfei Huang, Shihong Ying, Meixiang Huang, Chenhui Qiu, Fang Lu, Zhiyi Peng, Dexing Kong

**Affiliations:** 1 School of Mathematical Sciences, Zhejiang University, Hangzhou 310027, China; 2 Department of Radiology, The First Affiliated Hospital, Zhejiang University School of Medicine, Hangzhou 310030, China; 3 The School of Mathematics and Statistics, Minnan Normal University, Zhangzhou 363000, China; 4 Department of Mathematics, Zhejiang University of Science and Technology, Hangzhou 310023, China

**Keywords:** nonlinear registration, multiphase liver CT image, dynamic subtraction, 3D voxel-wise assessment

## Abstract

Voxel-wise quantitative assessment of typical characteristics in three-dimensional (3D) multiphase computed tomography (CT) imaging, especially arterial phase hyperenhancement (APHE) and subsequent washout (WO), is crucial for the diagnosis and therapy of hepatocellular carcinoma (HCC). However, this process is still missing in practice. Radiologists often visually estimate these features, which limit the diagnostic accuracy due to subjective interpretation and qualitative assessment. Quantitative assessment is one of the solutions to this problem. However, performing voxel-wise assessment in 3D is difficult due to the misalignments between images caused by respiratory and other physiological motions. In this paper, based on the Liver Imaging Reporting and Data System (v2018), we propose a registration-based quantitative model for the 3D voxel-wise assessment of image characteristics through multiple CT imaging phases. Specifically, we selected three phases from sequential CT imaging phases, i.e., pre-contrast phase (Pre), arterial phase (AP), delayed phase (DP), and then registered Pre and DP images to the AP image to extract and assess the major imaging characteristics. An iterative reweighted local cross-correlation was applied in the proposed registration model to construct the fidelity term for comparison of intensity features across different imaging phases, which is challenging due to their distinct intensity appearance. Experiments on clinical dataset showed that the means of dice similarity coefficient of liver were 98.6% and 98.1%, those of surface distance were 0.38 and 0.54 mm, and those of Hausdorff distance were 4.34 and 6.16 mm, indicating that quantitative estimation can be accomplished with high accuracy. For the classification of APHE, the result obtained by our method was consistent with those acquired by experts. For the WO, the effectiveness of the model was verified in terms of WO volume ratio.

## 1. Introduction

**Figure 1:**
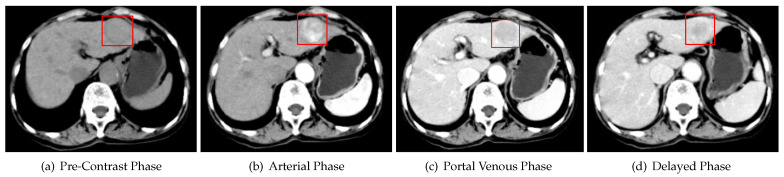
Four-phase image of CT images. Lesion area is labeled by the red box: (**a**) shows the pre-contrast phase image without injecting intravenous contrast media; (**b**) shows the lesion areas tend to enhance more strongly than background liver during late arterial phase imaging (APHE); (**c**) shows the liver continues to enhance, and the lack of portal venous blood supply to HCCs results in the characteristic washout in the portal venous phase (WO); (**d**) shows the hypodense appearance of HCC in the delayed phases (WO).

Hepatocellular carcinoma (HCC) is the most common primary malignancy in the liver and the third leading cause of cancer-related death worldwide, causing more than 700,000 deaths every year [1,2,3]. Thus far, several of its imaging characteristics can be used for non-invasive diagnosis. Specifically, during hepatocarcinogenesis, the source of blood supply of the lesion shifts from a portal vein-predominant supply to a dominant arterial supply with venous drainage, whereas the normal liver receives approximately 25% of its blood supply from the hepatic artery and 75% from the portal vein. After contrast media (CM) injection, given that HCCs receive blood mainly from the hepatic arteries, they are enhanced more strongly than background liver during imaging in the arterial phase (AP), a phenomenon known as AP hyperenhancement (APHE). The normal liver then continues to enhance during the contrast-enhanced portal venous phase (PV). However, the lack of portal venous blood supply to HCCs results in its characteristic washout (subsequent washout, WO) in the PV, especially in the delayed phase (DP). In this paper, we demonstrate these different imaging characteristics in multiphase computed tomography (CT) images, which are usually employed for detection and diagnosis [4,5,6,7], see Figure 1. Compared with background liver, the area marked with a red box is clearly hyperdense in the AP, i.e., APHE, hypodense in the PV, and hypodense on DP images, i.e., WO. Given a situation where the region is isodense in the PV, the pre-contrast phase (Pre), AP, and DP images were selected for our study.

**Figure 2:**
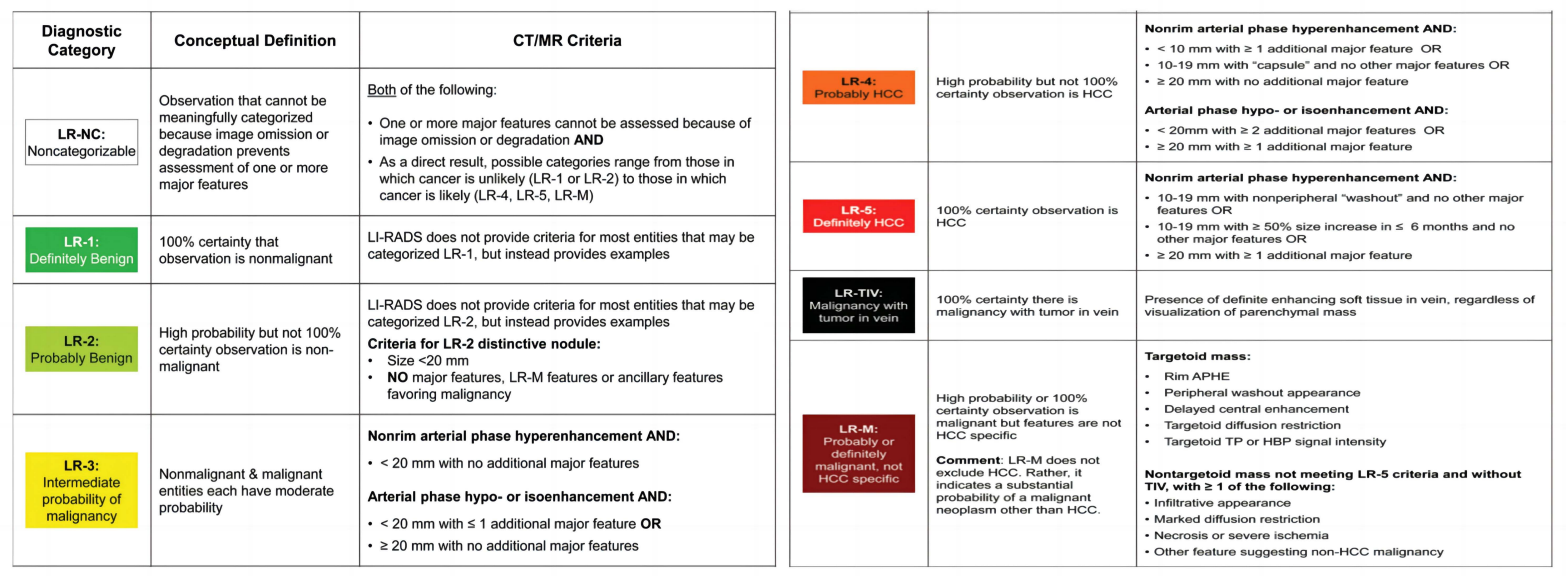
Summary of CT and MRI diagnostic Liver Imaging Reporting and Data System (LI-RADS) categories. APHE = arterial phase hyperenhancement; HBP = hepatobiliary phase; HCC = hepatocellular carcinoma; TIV = tumor in vein; TP = transitional phase [11].

Based on typical image characteristics, particularly APHE and WO [8,9], which reflect the probability of HCC or the presence of malignancy in veins, the American College of Radiology first introduced the Liver Imaging Reporting and Data System (LI-RADS) in 2011, which was updated in 2018 (LI-RADS v2018) [10,11,12], to provide the standardized qualitative reporting of liver lesions in CT and magnetic resonance imaging (MRI) in a noninvasive diagnostic manner for patients at risk for HCC. In accordance with the eight unique diagnostic categories (Figure 2) defined by LI-RADS, radiologists first routinely examine multi-phase liver CT to estimate features, i.e., using two-dimensional (2D) slice images to visually search for corresponding 2D regions of interest (ROIs) between 3D CT volumes to qualitatively estimate features and then determine the diagnostic category for these data by stepwise comparison of relevant imaging characteristics [11], i.e., giving a diagnostic result.

However, a weakness of LI-RADS remains, namely, the subjectivity of qualitative image feature interpretation, which will potentially affect inter-reader agreement and ultimately diagnostic accuracy.

One of the solutions to overcome the weakness of LI-RADS is to investigate the quantitative estimation of major imaging features. To date, several research studies have focused on quantitative assessment of HCC features in contrast-enhanced liver CT [13,14] and MRI [15,16,17]. For the APHE, Kim et al. [13] defined a quantitative color mapping of the arterial enhancement fraction, which can increase the sensitivity and diagnostic performance of multiphasic multidetector CT for detecting HCC. They also incorporated a diffemorphic registration model, but to our knowledge, most of the approaches used to register medical images assume that the entire deformation field should be smooth, which contradicts the feature of deformation field between multi-phase CT images due to the sliding motion on the liver; as a result, the registration problem becomes more complicated, that is, the discontinuous deformation field between the abdominal organs and the abdominal–thoracic wall. In addition, such approaches lack quantitative assessment of WO, which is also important for diagnosis. For the WO, Liu et al. [14] set a threshold value (≥107) for defining WO after calculating the percentage decay ratio, which correlated well with pathologically proven diagnoses of HCC in explanted livers. In [15], Agarwal et al. defined the WO as a 10% or greater decrease in signal intensity from 8 min to 20 min on 3D gradient echo images and utilized to distinguish hemangiomas from metastases on liver MRI, which can increase the accuracy of differentiating hemangiomas from metastases in gadoxetate disodium-enhanced MRI. Kloeckner et al. [16] quantitatively assessed WO in focal liver lesions using MRI and reported that the quantitative measure can provide more objective information and support in the diagnosis of HCC. Stocker et al. [17] compared the qualitative and quantitative results of APHE and WO appearance defined by LI-RADS v2018 in MRI; they reported that for WO quantitative assessment required further improvement to match the diagnostic accuracy of qualitative LI-RADS v2018. However, these WO-focused studies were performed in 2D ROIs selected by the radiologist to describe the imaging feature between the lesion and liver parenchyma. During selection, the radiologist must ensure that the position of the ROI is as consistent as possible across the corresponding phases. However, the following problems remain: (1) deformation: different breathing levels and other physiological movements during imaging will cause deformation between four-phase images; (2) 2D calculation: the information of the whole lesion is ignored, but only a part of the 2D area is selected for estimation; (3) inconsistency: for different radiologists or the same physician, the results of feature evaluation may be different at various times.

### Contributions and Organization

To solve the above mentioned problems, we propose a registration-based 3D voxel-wise quantitative model. The contributions are summarized as follows:

- Piece-wise smooth nonlinear registration: an iterative reweighted local cross-correlation (IRLCC) method was used to overcome the deformation caused by various reasons, and it assumes that the entire deformation is piece-wise smooth.
- Three-dimensional calculation: imaging features were quantified voxel-wise, based on registered images.
- Consistency: all 3D information of the whole lesion was used for quantitative analysis and visualization.

**Figure 3:**
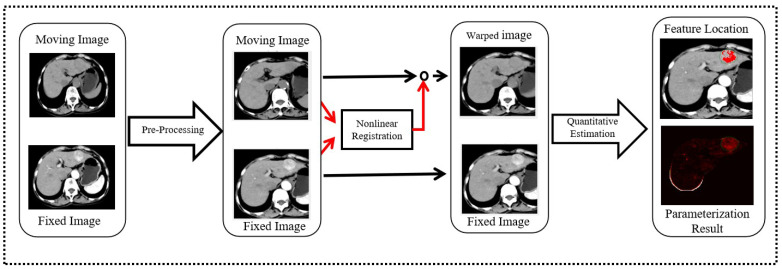
Illustration of 3D quantitative estimation model. Circle denotes a composition where the warped image is reconstructed by the deformation field computed from nonlinear registration, resulting in the warped image.

In addition, we improved the quantitative assessment of WO by extracting the feature position based on its definition in LI-RADS and then calculating the corresponding quantitative values. Figure 3 shows the flowchart of the entire process in this paper, which takes two specific phases of images as an example, starting with image preprocessing, such as liver segmentation, resampling, cropping, intensity normalization, and rigid registration. Then, Pre and DP CT images were registered to the AP CT images to deal with the misalignments caused by respiratory and other physiological motions during different CT image acquisition phases. Finally, a 3D voxel-wise quantitative assessment model was used to calculate the value of each voxel to describe APHE and WO.

The remainder of this study is organized as follows. Section 2 describes in detail our proposed registration-based 3D voxel-wise quantitative assessment model of the major imaging features of HCC. Section 3 provides the parameter settings involved in the registration and the corresponding experimental results. Section 4 offers the discussion of results and further research ideas. Section 5 provides the conclusions.

## 2. Materials and Methods

Images are acquired at different stages after the injection of the CM. The patient must hold his or her breath during the imaging. However, patients usually cannot hold their breath in the same respiratory position in different phases of image acquisition. Thus, the liver and its diseased areas appear as deformations in different phase images due to significant displacement in the cranio-caudal direction caused by respiratory movements and distortion caused by diaphragm and rib movements. The entire deformation is highly discontinuous due to the sliding movement on the liver. In this context, we introduced a nonlinear registration model to handle deformations, and this model uses a robust function to obtain a piecewise smooth transformation and then voxel-wise quantify features in 3D based on the aligned three-phase image.

### 2.1. Dataset

This retrospective study considers 35 multiphase CT images of patients who underwent liver resection for HCC between 2014 and 2015. All data were pathologically proven to be HCC. Liver CT examinations were performed by one of two manufacturers (Toshiba Medical Systems, Tochigi, Japan, and Philips, Eindhoven, The Netherlands) at the First Affiliated Hospital of Zhejiang University. Typical scan parameters were as follows: 120–150 mL iodinated contrast (iopamidol 300–370 mg iodine/mL) was power injected intravenously at a rate of 2.5–3.0 mL/s. AP, PV, and DP images were acquired at 33, 65, and 100 s after CM injection.

Table 1 provides the basic information of the clinical dataset, including the distribution of gender, age, slice thickness, and slice plane resolution. The mean age of the patients was 60 years old. Clinically, the tumor with the maximum diameter larger than 5 cm is the greater tumor, and that less than 3 cm is the small tumor [18]. Therefore, the maximum diameters of the tumors in the dataset were divided into three categories.

### 2.2. Image Registration Framework

The first part of the quantization model, which uses an image registration framework to recover the deformation caused by various reasons to improve the accuracy of quantification, consists of two steps: (1) image preprocessing and (2) nonlinear registration. In image preprocessing, the liver in each phase image was semi-automatically segmented, and the lesion area was marked by experts. In addition, we employed rigid registration to roughly match the images. The nonlinear registration was then used to estimate the final transformation with piecewise smoothing and volume-preserving constraints.

#### 2.2.1. Image Preprocessing

Prior to registration, several critical pre-processing steps were performed as follows. First, semi-automatic segmentation of the liver was performed for each phase image in accordance with the literature [19], and the lesion region of each phase image was outlined by experts to ensure that the shape and volume of the region were as consistent as possible. The images were then resampled to a new voxel spacing of 1×1× mm${}^{3}$, and liver segmentation was used to find a bounding box to crop unnecessary areas around the target object. Next, the intensity of the images was normalized to [0,1] using the window center and window width. Finally, rigid pre-alignment based on the normalized correlation coefficient metric was performed using SimpleITK  [20,21,22].

#### 2.2.2. Nonlinear Registration: The IRLCC Method

Given the large amount of local deformations between the different phase images caused by respiratory and other physiological movements, rigid pre-registration of the images alone did not meet the high accuracy requirements of the subtraction operation (quantitative assessment) [11]. Therefore, we introduced a nonlinear registration method to capture local deformation; it is called the IRLCC method, and it was proposed in our previous work [23]. Let the fixed and moving images, denoted by $I_{1}:R^{3}\to R$ and $I_{2}:R^{3}\to R$ with compact supporting domain $\Omega \subset R^{3}$, be continuously differentiable. The task of image registration is to find a reasonable spatial correspondence $T:\Omega \to R^{3}$ between $I_{2}\left(x\right)$ and $I_{1}\left(x\right)$ that makes the deformed moving and fixed images as similar as possible. In general, the optimal spatial correspondence is given by minimizing the following energy functional:

(1) $$L\left(T\right)=D\left(I_{1}\left(x\right),I_{2}\left(T\left(x\right)\right)\right)+\alpha R\left(T\left(x\right)\right)+\beta P\left(I_{1}\left(x\right),I_{2}\left(T\left(x\right)\right)\right),$$

where D(·) is a similarity measure that describes the difference between the deformed moving and fixed images, R(·) is a regularization term that penalizes irregular deformations,  and P(·) is a priority constraint on images to make the result more reasonable for real applications. Moreover, α is a regularization parameter, β is a priority constraint parameter, and they balance the minimization of image distance, smoothness of deformation, and priority information of images.

Specifically for the deformation model, the IRLCC method estimates the displacement field $u:\Omega \to R^{3}$ to deform the moving image, i.e., T(x)=x+u(x). For the similarity measure, the intensity corresponding to the anatomical location was inconsistent across imaging stages due to the use of contrast agents but satisfies a potential functional relationship, which in this paper we assume can be approximated by a local linear function; in addition, the local cross-correlation [24,25,26], described as the first term in Equation (2), satisfies the local linearity assumption, and thus, we selected it as the similarity measure. On the other hand, given that the sliding motion on the liver leads to high discontinuity in the whole displacement field, and the different organs surrounding the liver lead to multiple motion, which disrupts the smoothness of the whole deformation, we considered a total variation-like regularization term [27,28,29] to describe a piecewise smooth deformation field, defined as the second term in Equation (2). In addition, the liver can be compressed globally in multiphase imaging, which led to a slight change in liver volume, but it can be easily compensated by a normalization step. Therefore, we incorporated the registration model with a global volume-preserving prior term [30,31], shown as the third term in Equation (2), to capture the local deformation of the liver while preserving liver volume during the registration process. Combining the above options, we registered multiphase liver CT images by optimizing the energy functional:

(2) $$L\left(u\right):=-\int_{\Omega }\frac{\upsilon_{12}{\left(x;u\right)}^{2}}{\upsilon_{1}\left(x\right)\upsilon_{2}\left(x;u\right)}dx+\alpha \int_{\Omega }\Phi (\sum_{i=1}^{3}|\nabla_{3}u_{i}{|}^{2})dx+\beta \int_{\Omega }\nabla \ell_{R_{p}}\cdot udx,$$

where $\upsilon_{12}\left(x;u\right)$, $\upsilon_{1}\left(x\right)$, and $\upsilon_{2}\left(x;u\right)$ represent the local covariance and variance of $I_{1}\left(x\right)$ and $I_{2}^{u}\left(x\right):=I_{2}\left(x+u\left(x\right)\right)$, respectively. $\Phi \left(x\right)=\sqrt{x^{2}+\epsilon^{2}}$, where ϵ=0.001. $\ell_{R_{p}}$ is the indicator function over the ROI in the moving image.

The local covariance and variances in Equation (2) are defined as follows:

(3) $$\begin{array}{cc} \upsilon_{1}\left(x\right) & =\frac{1}{|\omega_{x}|}\int_{z\in \omega_{x}}I_{1}{\left(z\right)}^{2}dz-{\left(\mu_{1}\left(x\right)\right)}^{2}dz,\\ \upsilon_{2}\left(x;u\right) & =\frac{1}{|\omega_{x}|}\int_{z\in \omega_{x}}I_{2}^{u}{\left(z\right)}^{2}dz-{\left(\mu_{2}^{u}\left(x\right)\right)}^{2}dz,\\ \upsilon_{12}\left(x;u\right) & =\frac{1}{|\omega_{x}|}\int_{z\in \omega_{x}}\left\{I_{1}\left(z\right)-\mu_{1}\left(x\right)\right\}\left\{I_{2}^{u}\left(z\right)-\mu_{2}^{u}\left(x\right)\right\}dz, \end{array}$$

where $\omega_{x}$ is the neighborhood of x set to 7×7×7, $\mu_{1}\left(x\right)=\frac{1}{|\omega_{x}|}\int_{z\in \omega_{x}}I_{1}\left(z\right)dz$ and $\mu_{2}^{u}\left(x\right)=\frac{1}{|\omega_{x}|}\int_{z\in \omega_{x}}I_{2}^{u}\left(z\right)dz.$

Evidently, the objective energy functional is highly nonconvex and nonlinear with respect to *u*, which will lead to a challenging optimization formulation. Faced with this situation, we applied the coarse-to-fine refinement scheme with a dense Gaussian pyramid (e.g., with a downsampling rate η=0.75) on the images, which can avoid falling into the local optimum of the above functional and is considered the first-discretize-then-optimize method to solve our model.

**Discretization and Optimization**—First, we discretized the domain Ω into a grid, used the grid directly to obtain discretized images, and then constructed a coarse-to-fine pyramid with Gaussian filtering of each discretized image. Let $I_{1}^{1}\left(x\right)\cdots I_{1}^{L}\left(x\right)$ be the L-level coarse-to-fine pyramidal representation of the fixed image $I_{1}\left(x\right)$ from the coarsest resolution $I_{1}^{1}\left(x\right)$ to the finest resolution $I_{1}^{L}\left(x\right)=I_{1}\left(x\right)$ and $I_{2}^{1}\left(x\right)\cdots I_{2}^{L}\left(x\right)$ be the L-level coarse-to-fine pyramidal representation of the moving image $I_{2}\left(x\right)$. At each level l(=1,⋯,L), let the deformation field $u^{l-1}\left(x\right)$ estimated from the previous level be the initial value. The deformation field $u^{l}\left(x\right)$ between two given images can be replaced by $u^{l}\left(x\right)=u^{l-1}\left(x\right)+h\left(x\right)$, where $h\left(x\right)=(h_{1}\left(x\right),h_{2}\left(x\right),h_{3}\left(x\right))$ is the incremental deformation. Therefore, the optimum deformation $u^{l}\left(x\right)$ was calculated via estimating the optimum incremental deformation. For the coarsest level, i.e.,  l=1. The initial previous-level deformation was set to **0**.

In accordance with the difference rule, the approximation of integrals and derivatives can be obtained, and the continuous functional in Equation (2) can be discretized as follows:

(4) $$\begin{array}{c} L\left(h\right)\;=\;-\sum_{x}LCC\left(x;u+h\right)+\sum_{x}{\alpha \Phi (|\nabla \left(u\left(x\right)+h\left(x\right)\right)|}^{2})+\sum_{x}\beta \nabla l_{R_{p}}\cdot \left(u\left(x\right)+h\left(x\right)\right), \end{array}$$

where we have omitted the superscript l−1 and *l* for convenience.

Let

(5) $$\begin{array}{c} A\left(x\right)\;=\;\sum_{y\in \omega_{x}}{\left(I_{1}\left(y\right)-\mu_{1}\left(x\right)\right)}^{2},\\ B\left(x;h\right)\;=\;\sum_{y\in \omega_{x}}{\left(I_{2}\left(y+u\left(y\right)+h\left(y\right)\right)-\mu_{2}\left(x;u+h\right)\right)}^{2},\;\\ C\left(x;h\right)\;=\;{\left(\sum_{y\in \omega_{x}}\left(I^{1}\left(y\right)-\mu_{1}\left(x\right)\right)\left(I^{2}\left(y+u\left(y\right)+h\left(y\right)\right)-\mu_{2}\left(x;u+h\right)\right)\right)}^{2},\\ I_{2}\left(x+u\left(x\right)+h\left(x\right)\right)\;\approx \;I_{2}\left(x+u\left(x\right)\right)\;+\;\nabla I_{2}\left(x+u\left(x\right)\right)\;\cdot \;h\left(x\right). \end{array}$$

then, we can derive the derivative $\frac{\partial L(h)}{\partial h_{1}},\frac{\partial L(h)}{\partial h_{2}},\frac{\partial L(h)}{\partial h_{3}}$ at x=(x,y,z), set it equal to 0, and rewrite it in a matrix form that omits the variables:

(6) $$\begin{array}{c} \left(\begin{array}{ccc} \frac{2C^{2}}{AB^{2}}{\left(I_{2x}\right)}^{2}+\alpha \dot{\Phi }\Delta & \frac{2C^{2}}{AB^{2}}I_{2x}I_{2y} & \frac{2C^{2}}{AB^{2}}I_{2x}I_{2z}\\ \frac{2C^{2}}{AB^{2}}I_{2x}I_{2y} & \frac{2C^{2}}{AB^{2}}{\left(I_{2y}\right)}^{2}+\alpha \dot{\Phi }\Delta & \frac{2C^{2}}{AB^{2}}I_{2y}I_{2z}\\ \frac{2C^{2}}{AB^{2}}I_{2x}I_{2z} & \frac{2C^{2}}{AB^{2}}I_{2x}I_{2z} & \frac{2C^{2}}{AB^{2}}{\left(I_{2z}\right)}^{2}+\alpha \dot{\Phi }\Delta \end{array}\right)\left(\begin{array}{c} h1\\ h2\\ h3 \end{array}\right)=\left(\begin{array}{c} b_{1}\\ b_{2}\\ b_{3} \end{array}\right), \end{array}$$

where Δ denotes the discrete Laplace operator based on the second-order difference formula, and $\dot{\Phi }$ denotes the derivative of Φ:

$$\begin{array}{c} \begin{array}{cc} & g_{t}=\left(I_{1}-\mu_{1}\right)-\frac{C}{B}\left(I_{2}-\mu_{2}\right),\\ & b_{1}=-\frac{2C}{AB}g_{t}I_{2x}+\alpha \dot{\Phi }\Delta u_{1}+\beta \ell_{R_{p_{x}}},\\ & b_{2}=-\frac{2C}{AB}g_{t}I_{2y}+\alpha \dot{\Phi }\Delta u_{2}+\beta \ell_{R_{p_{y}}},\\ & b_{3}=-\frac{2C}{AB}g_{t}I_{2z}+\alpha \dot{\Phi }\Delta u_{3}+\beta \ell_{R_{p_{z}}}. \end{array} \end{array}$$

Finally, we vectorized $h_{1}$, $h_{2}$, and $h_{3}$ into $H_{1}$, $H_{2}$, and $H_{3}$, respectively, and used the following fixed-point iteration method Algorithm 1, which includes the outer iteration (OutIter) and inner iteration (InIter) to solve Equation (6).
**Algorithm 1:** Fixed-Point Iteration Algorithm**Input**: $I_{1}^{l},I_{2}^{l},U^{l-1}$, l is the current level.**Output**: $U^{l}$.Initialization: Upsample $U^{l-1}$ to the size in level l, then deform the moving image: $I_{2}^{l}\left(x\right)=I_{2}^{l}\left(x+U^{l-1}\left(x\right)\right)$;
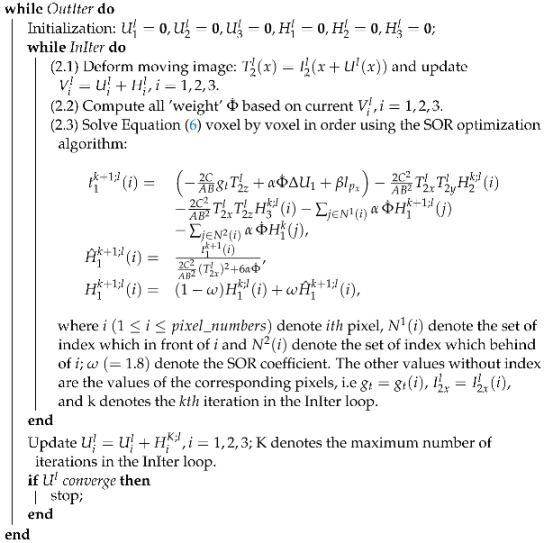


### 2.3. The 3D Voxel-Wise Quantitative Assessment of Imaging Features

As the second part of the quantitative model, we introduced a novel 3D voxel-wise quantitative evaluation criterion (extraction location then quantification) of the two main imaging features in HCC, inspired by the features measurement in LI-RADS v2018. The measurement of such features at each image location was performed by evaluating the local intensity changes across different CT imaging phases through the subtraction operation.

#### 2.3.1. Locations Extraction

The first step of the quantitative method is to extract feature locations because determining a threshold to where the feature is difficult when the feature is estimated directly. Specifically, the locations of two main features were extracted by the following operations.

**Operations of extracting APHE location: Equations (7) and (8)**—According to the definition of APHE in LI-RADS v2018, the area must meet two conditions: (1) its enhancement in AP must be unequivocally greater in whole or in part than the liver, and (2) the enhanced part must be brighter than the liver in AP. In this section, we use two subtraction operations to describe these conditions:

(7) $$\begin{array}{c} \left\{\begin{array}{c} B_{ap}=M_{ap}-LM_{ap},\\ Sub_{1}=M_{ap}-M_{pre},\\ E_{sub_{1}}=sub_{1}-LM_{sub_{1}},\\ mask_{1}\left(x\right)=\left\{\begin{array}{c} 1,\;E_{sub_{1}}>\;0\;\;\;\mathrm{condition}\;\left(1\right)\\ 0,\;\mathrm{otherwise} \end{array}\right.,\\ mask_{2}\left(x\right)=\left\{\begin{array}{c} 1,\;B_{ap}\left(x\right)>\;0\;\;\;\mathrm{condition}\;\left(2\right)\\ 0,\;\mathrm{otherwise} \end{array}\right., \end{array}\right. \end{array}$$

where $LM_{ap}$ and $LM_{sub_{1}}$ represent the adjacent liver values in the image matrix $M_{ap}$ and $sub_{1}$, respectively. $M_{ap}$ and $M_{pre}$ represent the warped Pre and AP liver image matrices, respectively.

By definition, the APHE location must satisfy values greater than zero in the $B_{ap}$ and $E_{sub_{1}}$ images. Thus, we can obtain the location of APHE, that is, $mask_{APHE}$, using Equation (8), where the value corresponding to the voxel with the APHE feature in $mask_{APHE}$ is 1.

(8) $$\begin{array}{c} mask_{APHE}\left(x\right)\;=\;mask_{1}\left(x\right)\wedge mask_{2}\left(x\right), \end{array}$$

where ∧ is the logical AND operation.

**Operations of extracting WO location: Equations (9)–(11)**—According to its definition in LI-RADS v2018, which is the visually assessed temporal reduction of enhancement of the area in whole or in part relative to the composite liver tissue from earlier to later phase, resulting in hypoenhancement in the post-AP, we divided the position extraction into two steps. One step is to check that the area is at least slightly enhanced in the AP (not necessarily APHE) by Equation (9).

(9) $$\begin{array}{cc} & mask_{3}\left(x\right)=\left\{\begin{array}{ccc} & 1, & \;sub_{1}\left(x\right)>\;0\;\;\;\\ & 0, & \;\mathrm{otherwise} \end{array}\right. \end{array}$$

The other step is to determine whether the area is darker than the liver in the DP image or the (DP−Pre) subtraction image by Equation (10) and then to extract the WO location $mask_{WO}$ by Equation (11).

(10) $$\left\{\begin{array}{c} B_{dp}\;=\;M_{dp}-LM_{dp},\\ sub_{2}\;=\;M_{dp}-M_{pre},\\ W_{sub_{2}}\;=\;sub_{2}-LM_{sub_{2}},\\ mask_{4}\left(x\right)=\left\{\begin{array}{c} 1,\;B_{dp}\left(x\right)>\;0\;\;\;\\ 0,\;\mathrm{otherwise} \end{array}\right.,\\ mask_{5}\left(x\right)=\left\{\begin{array}{c} 1,\;W_{sub_{2}}\left(x\right)>\;0\;\;\;\\ 0,\;\mathrm{otherwise} \end{array}\right.,\\ mask_{DP}\left(x\right)\;=\;mask_{4}\left(x\right)\vee mask_{5}\left(x\right), \end{array}\right.$$

(11) $$\begin{array}{c} mask_{WO}\left(x\right)\;=\;mask_{3}\left(x\right)\wedge mask_{DP}\left(x\right), \end{array}$$

where $M_{dp}$ is the warped DP image matrix. $LM_{dp}$ and $LM_{sub_{2}}$ represent the adjacent liver value in image matrix $M_{dp}$ and $sub_{2}$, respectively, and ∨ is the logical OR operation.

**Operation of calculating adjacent liver value: Equation (12)**—Given that the extraction of APHE and WO location depends on comparison with the surrounding liver value, their value must be obtained. In particular, we first determined the liver area using the liver mask containing lesion and normal liver. Then, we removed the lesion area and calculated the mean (μ) and variance (σ) of the intensity corresponding to the remaining part. Finally, we gave the adjacent liver value as follows:

(12) $$LM=\left\{\begin{array}{cc} \mu +2\times \sigma , & \;\mathrm{if}\;\mathrm{the}\;\mathrm{operations}\;\mathrm{is}\;\mathrm{used}\;\mathrm{to}\;\mathrm{extract}\;\mathrm{APHE}\;\\ \mu -2\times \sigma , & \;\mathrm{if}\;\mathrm{the}\;\mathrm{operations}\;\mathrm{is}\;\mathrm{used}\;\mathrm{to}\;\mathrm{extract}\;\mathrm{WO}\; \end{array}\right..$$

**Add Operation: Equation (13)**—To show the APHE and WO features simultaneously, we logically combined $mask_{APHE}$ and $mask_{WO}$:

(13) $$S\left(x\right)=mask_{APHE}\left(x\right)\wedge mask_{WO}\left(x\right).$$

#### 2.3.2. Estimation

The second step was to quantitatively estimate these features by assigning values to each voxel; it uses the feature position mask to quantify the features in the region voxel-wise.

To represent the degree of APHE, our model calculates the percentage of arterial enhancement (PAE) [17] for the corresponding voxels as follows:

(14) $$PAE\left(x\right)=\frac{sub_{1}\left(x\right)}{M_{pre}\left(x\right)}.$$

**Figure 4:**
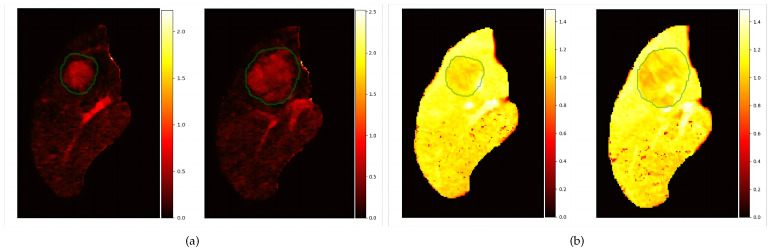
The parameterization results: (**a**) shows two slices of APHE results and (**b**) shows two slices of WO results. The green contour is the lesion contour in the AP image.

To describe the degree of WO, we improved the lesion-to-liver contrast ratio (LLCR), which is similar to that in [17] but slightly more complicated. We needed to ensure that the voxel is in $mask_{4}$ or $mask_{5}$. Figure 4 illustrates the typical quantitative results.

(15) $$LLCR_{0}\left(x\right)\;=\;\left\{\begin{array}{ccc} \frac{-B_{dp}\left(x\right)}{LM_{dp}}, & \; & \;mask_{4}\left(x\right)=\;1\\ \frac{-W_{sub_{2}}\left(x\right)}{LM_{sub_{2}}}, & \; & \;mask_{5}\left(x\right)=\;1\\ 0, & \; & \;Otherwise \end{array}\right..$$

In summary, we can extract the location of features and quantify features voxel-wise using Equations (7)–(15). Utilizing these location masks, we can calculate the volume over features and lesion areas according to Equation (16). In addition, the ratio *R*, defined as Equation (16), can be calculated and used to quantify how much the area of the features accounts for the lesion area. Using the quantified results, the areas with features can be displayed as a heat map.

(16) $$\begin{array}{c} \left\{\begin{array}{c} V_{feature}\;=\;\sum_{x\in \Omega_{lesion}}mask_{feature}\left(x\right),\\ V_{lesion}\;=\;\sum_{x\in \Omega }mask_{lesion}\left(x\right),\\ R\;=\;\frac{V_{feature}}{V_{lesion}}, \end{array}\right. \end{array}$$

where feature can be APHE or WO.

## 3. Results

In this section, we first show the parameter settings of the registration experiments. Then, the results of the registration model are presented quantitatively and visually, and the voxel-wise quantitative findings are displayed in 3D volume and 2D slices to visualize the location of features and their corresponding values. Finally, the quantitative results are used to classify the two features for quantitative analysis.

### 3.1. Setting of Registration Parameters

Table 2 and Table 3 list the hyperparameters involved in the experiments, which were selected based on experimental results. The number of levels can be calculated adaptively based on the size of the input image, the given size of the coarsest level image, and the downsampling factor η (=0.75).

### 3.2. Registration Results

We performed experiments with three criteria metrics, namely, the dice similarity coefficient (DSC, [32]), mean surface distance (MSD, [33]), and Hausdorff distance (HDD, [34]), to evaluate the registration accuracy, and the results of the metrics are summarized in Table 4. (1) Given that the pre and DP CT images were each registered to the AP CT image by the nonlinear registration framework, the results of the registration experiment were also evaluated separately. (2) With the liver registration, we finally registered images according to the ROI defined by the lesion mask to achieve accurate lesion area matching. In general, the larger the value of DSC and the smaller the value of MSD and HDD, the better the effect of the algorithm.

Table 4 shows that after nonlinear registration, the DSC of the liver mask increased, and the MSD and HDD of the liver surface decreased. Similarly, the DSC increased, and the MSD and HDD of the lesion area decreased. Specifically, for the registration of Pre and AP images, the mean of liver DSC was 98.6%, and the means of liver surface MSD and HDD were 0.38 and 4.43 mm, respectively. In addition, the mean of lesion DSC was 98.7%, the means of lesion MSD and HDD were 0.31 and 2.24 mm, respectively. For the registration of DP and AP images, the mean of liver DSC was 98.1%, the means of liver surface MSD and HDD were 0.54 and 6.16 mm, respectively. Moreover, the mean of lesion DSC was 98.3%, the means of the lesion surface MSD and HDD are 0.64 mm and 2.34 mm, respectively. Compared with the results of no and rigid registration, the proposed registration framework significantly improved the accuracy.

**Figure 5:**
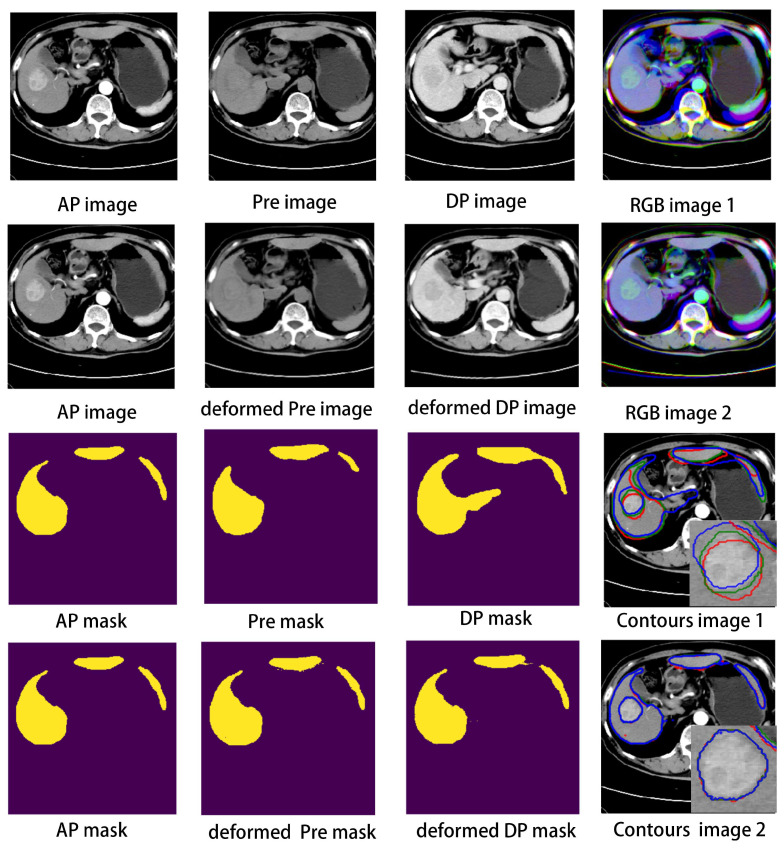
Registration illustration: RGB image shows AP, Pre, and DP image together without or with deformation. Contours image shows three mask contours on AP image without or with deformation.

**Figure 6:**
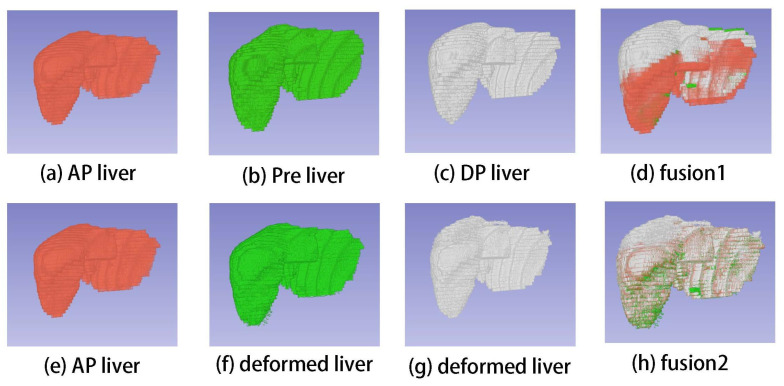
Three-dimensional view: (**a**–**c**) are fused together to show in (**d**); (**f**) shows deformed Pre liver, (**g**) shows deformed DP liver; and (**e**–**g**) are fused together to show in (**h**).

The performance of the proposed registration framework was also displayed in visual view. Figure 5 shows the three phase images with the corresponding liver and lesion contours in registration and non-registration forms. Compared with the non-registered results in RGBimage1, the registered results in RGBimage2 showed good visual agreement. Furthermore, the liver and lesion contours in Pre, AP, and DP (ContourImage2) achieved a high degree of consistency after nonlinear registration. Figure 6 shows livers in 3D volume view; subfigure (d) shows the three-phase liver volumes together and the many misalignments between them, whereas subfigure (h) reveals the evidently reduced misalignments. All the results indicate that our registration framework can achieve high accuracy, which means that we can use the subtraction to extract the feature location.

### 3.3. Voxel-Wise Quantitative Assessment Results and Visualization

Applying Equations (7)–(15) to the registered three-phase images, we can obtain the voxel-wise quantitative assessment of the two main imaging features.

Figure 4 shows the voxel-wise quantitative assessment results of APHE and WO. From Figure 4a, the enhancement of the lesion area in AP was unequivocally greater in part than that in the liver. In Figure 4b, several areas are darker than the liver in the DP source images or (DP—Pre) subtraction images.

**Figure 7:**
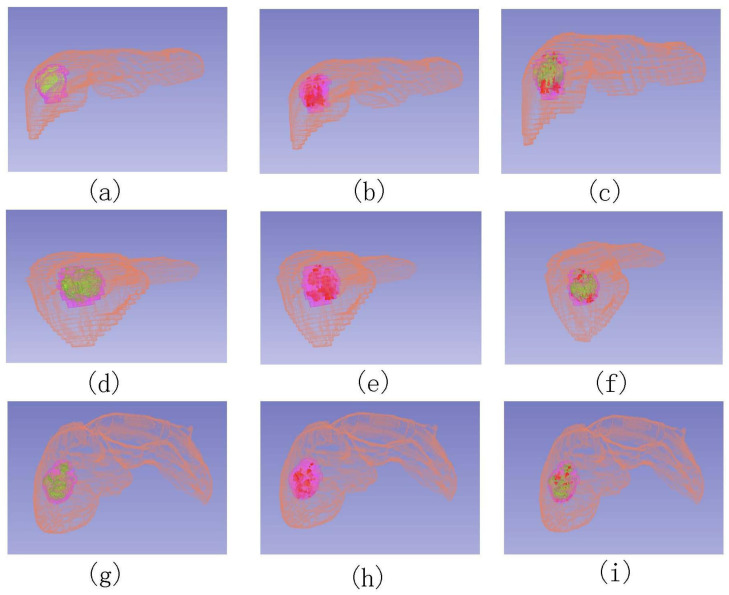
Locations of three different view: (**a**,**d**,**g**) show the APHE location (green), (**b**,**e**,**h**) show WO location (red), and (**c**,**f**,**i**) show the locations together. The purple area is the lesion delineated by experts.

Figure 7 shows the 3D feature location with 3 different views. From subfigures (a,d,g) and (b,e,h), not all parts of the lesion area marked by the experts had the two features, i.e., the lesion area contained normal liver tissue, which can be removed by the two feature masks to obtain a more accurate lesion area. As shown in (c,f,i), the WO area was not necessarily included in the APHE area in accordance with the definition of the two features.

**Figure 8:**
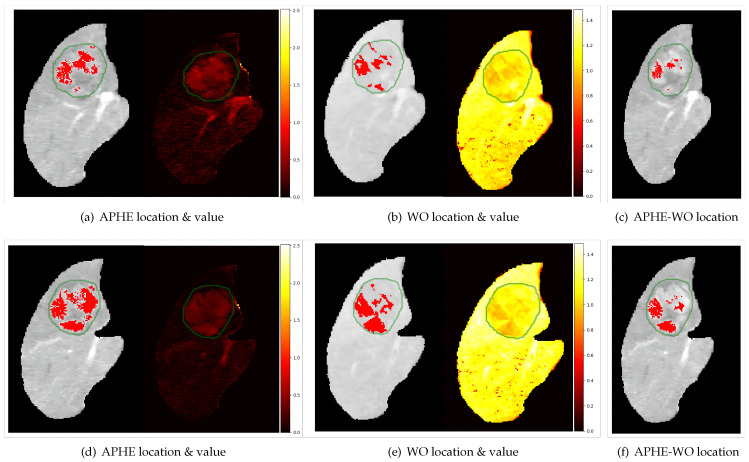
Images (**a**–**c**) are the same slice and (**d**–**f**) are the other same slice. The locations are shown on the AP liver image, and the heat map displays the corresponding quantitative estimation results, where the green contour is the lesion contour.

Figure 8 shows the locations of the features on the AP livers and the corresponding quantified heat map. In subfigures (a,d), the APHE value corresponding to the extraction position was unequivocally greater than the value corresponding to the surrounding liver, which confirmed the correctness of the extraction position. From figures (b,e), the value corresponding to WO was smaller than that of the surrounding liver, which can be used to verify the correctness of the extracted location. Subfigures (c,f) show the locations with both features. The figures reveal that not all regions with APHE in the AP showed WO in the DP, but regions that exhibited WO in the DP must have a certain degree of enhancement in the AP, which is consistent with the definition of the two features in LI-RADS.

### 3.4. Quantitative Analysis

**Figure 9:**
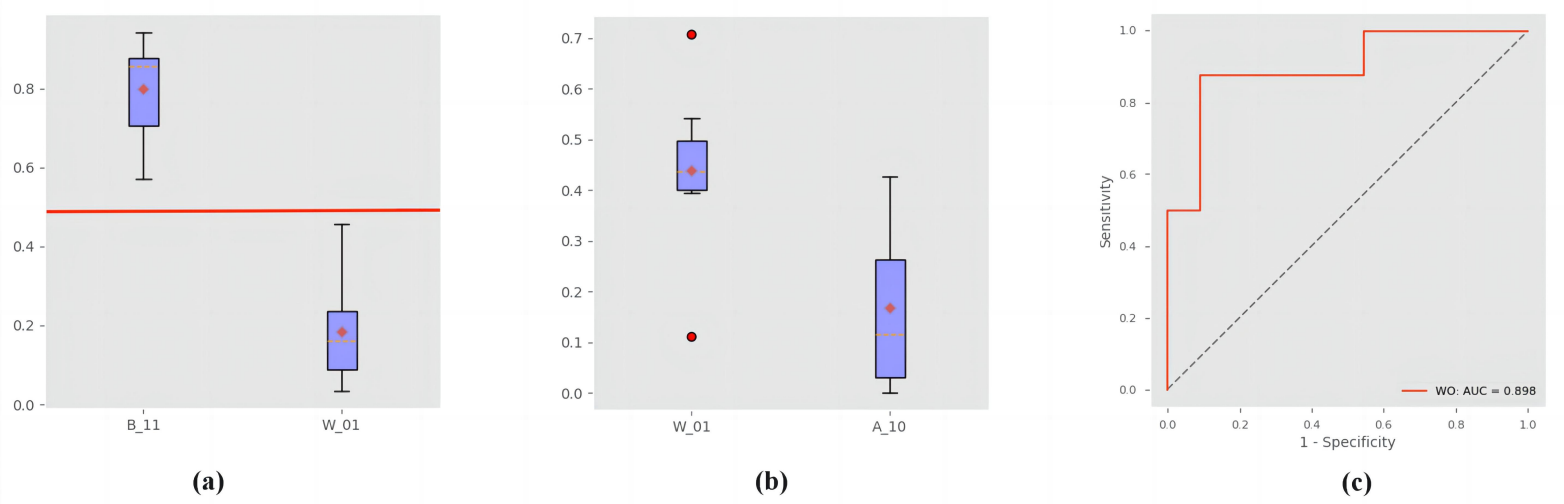
Classification results: (**a**,**b**) show the boxplot of APHE and WO volume ratio, respectively; (**c**) shows the ROC curve of WO volume ratio.

Experts first divided the data into three categories based on the features: $B_{11}$ (with two features), $A_{10}$ (only has APHE), and $W_{01}$ (only has WO). Then, we registered the data in accordance with the proposed method and calculated the corresponding feature volume ratio *R* based on the voxel-wise quantitative assessment results. Finally, we used the volume ratio *R* to classify these data, and the results are shown in Figure 9. Figure 9a shows the distribution of the APHE volume ratio of $B_{11}$ and $W_{01}$. No adjacent areas were observed, and a significant difference (Student *t*-test; $p=4.3\times 10^{-5}<0.01$) was detected in the two distributions. Thus, for the classification of APHE, the result obtained by the proposed method was consistent with that obtained by experts. Figure 9b shows the distribution of the WO volume ratio of $W_{01}$ and $A_{10}$. A significant difference was observed (Student *t*-test; $p=3.6\times 10^{-3}<0.01$), but several adjacent areas were detected in the two distributions. Figure 9c shows the receiver operating characteristic (ROC) curve of WO volume ratio; the area under the curve (AUC) was 89.8%. For the classification of WO, the results were consistent with those in [17] Stocker et al. (2020). The quantitative model needs further improvement to reach the diagnostic accuracy of the qualitative LI-RADS.

## 4. Discussion

In this paper, we mainly studied the voxel-wise assessment of two imaging features (APHE and WO) of HCC to reduce the subjective and qualitative interpretation of images by experts. Compared with the general method (visual estimation of features by 2D slices) used by radiologists, our method can provide a 3D quantified result, including the 3D feature location (Figure 4) and the corresponding quantitative map (Figure 4 and Figure 8) to assist them in making faster and more accurate diagnoses. In addition, the inter-reader agreement can be improved because the quantitative map, once calculated, can be considered fixed and independent of the user.

**Figure 10:**
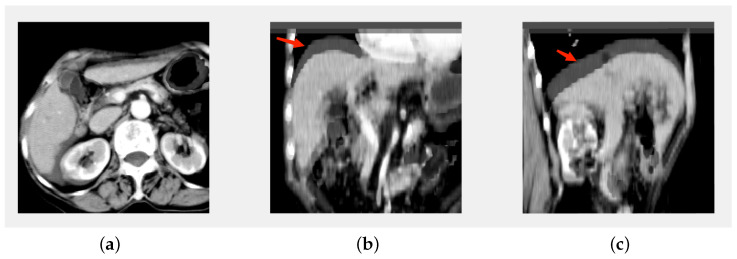
Illustration of sliding and deformation by superimposing the PV image onto the AP in axial (**a**); frontal (**b**); and sagittal view (**c**); respectively.

Compared with the quantitative method mentioned in the introduction, although [13] employed a registration step before 3D quantification, which assumes that the transformation is diffeomorphic and the liver is incompressible, a sliding motion occurs on the liver (Figure 10), which makes the registration problem more complicated, namely, the discontinuous deformation field between the abdominal organs and the abdominal–thoracic wall [35]. Our registration model uses the robust function Φ(x) to regularize a piecewise-smooth deformation field. As shown in Figure 6d,f, the deformation field can better recover the sliding motion.

Furthermore, these methods were first quantified, and the features were extracted using a threshold, i.e., in studies [36,37], quantitative APHE was defined as PAE≥0.1, which implies an intensity increase of at least 10% from the Pre to the AP, but the choice of threshold depends on the statistics of a large amount of data. However, from {Figure 8a,d, not all of the area in the lesion, whose corresponding quantitative value is larger than the surrounding liver, is the APHE, which makes the selection of threshold difficult and time-consuming. From Figure 8b,e, the area that showed WO in the DP must have a certain degree of enhancement in the AP. This phenomenon is consistent with the description of [17], which reported that “WO was only considered when the lesion had a PAE≥0 in the arterial phase, meaning that the lesion was iso- or hyperintense compared with the liver”. Thus, compared with selecting a threshold, our model directly extracts the feature location based on its definition in LIRADS v2018, avoids the error in selecting the threshold, and is more accurate and convenient. In addition, our method uses all the 3D information of the entire lesion for quantitative analysis and visualization, whereas other methods were applied quantitatively over the ROI. For example, Ref. [16] WO was studied quantitatively by placing one 25 mm${}^{2}$ ROI over each nodule and two 25 mm${}^{2}$ ROIs over adjacent liver parenchyma, using only part of the lesion area for quantitative estimation.

As shown in Figure 7, our method obtains a 3D area of two features, that is, the method can be used to detect features and give a relative area. However, the area may not be smooth. In the future, we will incorporate a post-processing method to deal with this issue to obtain a smooth segmentation mask of the lesion and integrate the quantitative assessment method into software to assist radiologists in diagnosing patients with or at risk for hepatocellular carcinoma. In addition, data will be collected and will be used to detect and classify the lesion based on the quantitative results and further for treatment evaluation, i.e., the quantified results can be used to calculate the volume of active tumors. Our model requires the segmentation of the liver area, and a low segmentation accuracy will lead to larger registration error. In this work, segmentation was implemented by a semi-automatic method, which is time-consuming. We will further investigate an automatic segmentation algorithm with high accuracy.

## 5. Conclusions

In this paper, we proposed a novel 3D voxel-wise quantitative criterion to evaluate imaging characteristics across multiple CT acquisition phases based on the LI-RADS, which was integrated with a piecewise smooth nonlinear image registration approach to address the misalignment problem in different phase images. The metrics and visual results showed the high accuracy of the registration framework. With the registered images, we can perform the operations to obtain quantitative assessment results. The visualization results demonstrated the effectiveness of the quantitative method for representing the two major imaging features (APHE and WO) of HCC. Figure 8 shows the features voxels on the fixed image (AP) with the quantitative assessment results. Compared with the original CT liver image, the figures can show the features region of HCC more clearly. From the application point of view, the operations involved in quantitative evaluation can be regarded as a method for detecting the APHE and WO features of HCC in 3D based on registered multiphase CT images. In the quantitative analysis, B11 and W01 have a significant difference and no adjacent areas from the distributions of APHE volume ratio, while W01 and A10 have a significant difference but several adjacent areas from the distributions of WO volume ratio. It shows the effectiveness of the quantitative evaluation method. Evidently, the 3D voxel-wise quantitative model of APHE and WO in HCC can be obtained with high accuracy using our method.

## Figures and Tables

**Table 1:** Patient and data information.

	Category	Number of Patients	Ratio (%)
Gender	Male	29	83%
	Female	6	17%
Age	≥60	23	66%
	<60	12	34%
maximum diameter	≥5 cm	8	23%
	3∼5 cm	21	60%
	<3 cm	6	17%
Thickness	5 mm		
Slice Resolution	512 × 512		
Number of slice	38∼53 slices		

**Table 2:** The parameters of nonlinear registration.

α	β	OutIter	InIter	SOR	Size of Coarsest Level Image
0.001	0.001	5	1	20	32

**Table 3:** The parameters of fine registration.

α	β	OutIter	InIter	SOR	Size of Coarsest Level Image
0.00015	0.0001	3	1	20	32

**Table 4:** Registration accuracy evaluated by DSC, MSD, and HDD (mean ± sd). The *N* refers to no registration, *R* to the rigid registration in the image preprocessing, and NR to the proposed nonlinear registration model with the volume preserving prior P(u) in Equation (2).

Region	Phase	Method	DSC (%)	MSD (mm)	HDD (mm)
liver	Pre–AP	*N*	89.2±5.7	2.64±1.42	11.80±3.45
		*R*	93.0±2.2	1.68±0.39	9.22±2.04
		*NR*	98.6±0.3	0.38±0.11	**4.34 ± 1.04**
	DP–AP	*N*	85.1±10.5	3.74±2.73	14.67±6.88
		*R*	90.1±6.0	2.18±1.27	10.72±4.37
		*NR*	**98.1 ± 1.2**	**0.54 ± 0.38**	**6.16 ± 2.92**
lesion	Pre–AP	*N*	67.5±11.5	3.04±1.76	10.13±3.89
		*R*	83.1±4.1	2.03±0.67	8.03±2.52
		*NR*	**98.7 ± 0.5**	**0.31 ± 0.27**	**2.24 ± 0.58**
	DP–AP	*N*	68.8±17.0	4.50±3.26	13.02±7.14
		*R*	80.9±8.6	2.42±1.32	9.00±3.61
		*NR*	**98.3 ± 0.8**	**0.64 ± 0.72**	**2.34 ± 0.82**

## Data Availability

Not applicable.

## Abbreviations

APHE	Arterial phase hyperenhancement
WO	Subsequent Washout
HCC	Hepatocellular carcinoma
AUC	Area under the curve
CT	Computed tomography
CM	Contrast media
MRI	Magnetic resonance imaging
Pre	Pre-contrast Phase
PAE	Percentage of arterial enhancement
AP	Arterial Phase
LI-RADS	Liver Imaging Reporting and Data System
PV	Portal Venous Phase
LLCR	Lesion-to-liver contrast ratio
DP	Delayed Phase
ROC	Receiver operating characteristics
ROIs	Regions of interests
